# Anticoccidial Resistance in *Eimeria* spp. From Thai Broiler Farms Using Shuttle Programs

**DOI:** 10.1155/vmi/8243240

**Published:** 2026-05-22

**Authors:** Suttitas Tongkamsai, Siraprapa Boobphahom, Rachan Apphaicha, Donruethai Sreta, Kriengwich Limpavithayakul, Niwat Chansiripornchai

**Affiliations:** ^1^ Faculty of Veterinary Medicine, Rajamangala University of Technology Tawan-ok, Chonburi, 20110, Thailand, rmutto.ac.th; ^2^ Research Center for Neuroscience, Institute of Molecular Biosciences, Mahidol University, Nakhon Pathom, 73170, Thailand, mahidol.ac.th; ^3^ Avian Health Research Unit, Department of Veterinary Medicine, Faculty of Veterinary Science, Chulalongkorn University, Bangkok, 10330, Thailand, chula.ac.th

**Keywords:** drug resistance, *Eimeria*, maduramicin, poultry, salinomycin

## Abstract

Coccidiosis is a widespread poultry disease caused by *Eimeria* species and significantly affects performance and productivity. In the field, co‐infections with multiple *Eimeria* species are commonly observed. While prophylactic coccidiostats are commonly used in feed for disease control, their extensive application has led to drug resistance, especially with the widespread use of shuttle programs in broiler production in Thailand. Understanding the resistance profiles of commonly used anticoccidials is crucial for developing effective prevention strategies. This study assessed the sensitivity of field isolates of *Eimeria acervulina* (*E. acervulina*)*, E. maxima*, and *E. tenella* from broiler farms in Thailand using four anticoccidial sensitivity tests (ASTs). A dose‐titration trial was conducted to determine effective inoculation doses, focusing on lesion induction and reduced weight gain while minimizing mortality. A total of 112‐day‐old broiler chicks were used to evaluate sensitivity to three widely used anticoccidial agents: narasin combined with nicarbazin, salinomycin, and maduramicin. Sensitivity assessment was conducted using four key indices: the anticoccidial index (ACI), the percentage of optimum anticoccidial activity (POAA), the reduction of lesion scores (RLS), and the relative oocyst production (ROP). During the study, broilers were fed the same combination of narasin + nicarbazin in the starter diet, while the in‐feed anticoccidial in the grower phase was either maduramicin or salinomycin. Following inoculation with oral doses of sporulated oocysts of *E. acervulina* (2 × 10^5^), *E. maxima* (1 × 10^5^), and *E. tenella* (1.5 × 10^4^), both medicated and infected‐unmedicated groups showed significantly lower weight gain compared to uninfected controls (*p* < 0.05). While both salinomycin and maduramicin treatments significantly reduced intestinal lesion scores and fecal oocyst shedding compared with the infected‐unmedicated group (*p* < 0.05), the reductions were incomplete. Notably, the calculated indices yielded discordant results: ACI and ROP values initially suggested sensitivity, whereas POAA and RLS indicated reduced efficacy. According to the study’s composite point system—where resistance in two indices signifies moderate resistance—the field isolates were classified as moderately resistant to both salinomycin and maduramicin. Histopathological analysis supported this, confirming that while medication reduced the severity of infection, it did not entirely prevent *Eimeria* colonization. This study provides the first systematic AST evaluation of Thai *Eimeria* isolates under shuttle‐program conditions. The findings highlight the emergence of moderate drug resistance even within these structured programs, emphasizing a critical practical relevance for the poultry industry. To sustain long‐term efficacy in Thai broiler production, there is an urgent need for strategic drug rotation and the integration of alternative control measures, such as vaccination and bio‐shuttle strategies.

## 1. Introduction

*Coccidia*, which are obligate intracellular protozoan parasites of the genus *Eimeria*, cause coccidiosis—a disease recognized as a significant threat to the poultry industry [1]. In poultry, the main etiological agents of coccidiosis are seven species of *Eimeria*: *E. tenella*, *E. acervulina*, *E. maxima*, *E. necatrix*, *E. mitis*, *E. brunetti*, and *E. praecox*. Chickens of various ages and breeds are susceptible to these *coccidia* [2]. Among these species, *E. tenella*, *E. acervulina*, and *E. maxima* are the most prevalent and can cause moderate to severe damage to the intestinal tract of chickens, according to reports worldwide [3, 4]. All these *Eimeria* strains are site‐specific; lesions are found in the intestines at specific locations for each *Eimeria* species. In chickens, *E. acervulina* infects the duodenum, and *E. maxima* infects the duodenum and jejunum, whereas *E. tenella* infects the caecum. This damage can lead to symptoms such as bloody diarrhea, decreased body weight gain (BWG), poor feed efficiency, and, in severe cases, death [5]. In commercial poultry systems, coccidiosis is typically managed using prophylactic coccidiostats in feed, with ionophore and synthetic drugs as the main control strategies. Ionophores, a type of polyether antibiotic, and synthetic compounds such as nicarbazin are commonly used to control the disease [6]. However, due to the prolonged use of anticoccidial drugs, significant drug resistance in *Eimeria* spp. Strains have gradually developed. This emergence of drug resistance in various regions around the world has diminished the efficacy of anticoccidials [7–10]. To mitigate resistance, Thai broiler production extensively uses shuttle programs that alternate drug classes within a single flock. Despite the widespread adoption of shuttle programs in the Thai poultry industry, the persistence of clinical and subclinical outbreaks suggests a gradual decline in anticoccidial efficacy. Current epidemiological data underscore this challenge, identifying *E. tenella* and *E. praecox* as the most prevalent species (40%) and revealing that multispecies co‐infections occur in 50% of positive cases. While these prevalence patterns are well documented, existing control strategies do not entirely preclude the emergence of resistance, particularly given the inherent risk of cross‐resistance between related drug classes. Furthermore, the increasing pressure to reduce or prohibit the continuous use of antimicrobials in food‐producing animals necessitates a more integrated approach to coccidial management [11]. Anticoccidial sensitivity tests (ASTs) must be conducted *in vivo*, which limits the selection of various drugs and *Eimeria* strains available for testing. Sensitivity testing involves infecting groups of medicated and nonmedicated birds with *Eimeria*. The parameters assessed in ASTs can include lesion scores, oocyst production measured as oocysts per gram (OPG), performance metrics such as BWG, mortality rates, or a combination of these factors, formulated into indices such as the anticoccidial index (ACI) [12, 13]. However, relying on a limited set of indices can lead to an incomplete assessment, particularly when field isolates exhibit discordant responses—where a drug may suppress clinical lesions but fail to control oocyst production. To ensure a more comprehensive and robust evaluation, this study integrates four distinct indices: ACI, percent of optimum anticoccidial activity (POAA), reduction of lesion scores (RLS), and relative oocyst production (ROP). While ACI and POAA prioritize clinical performance and survival, RLS and ROP provide direct insight into the parasite’s biological suppression. By utilizing this four‐dimensional framework, the study minimizes the risk of overestimating drug efficacy. It provides a more accurate characterization of the resistance profiles of strains managed under intensive shuttle medication regimens [14]. Despite the clinical importance of monitoring drug efficacy, ASTs are not yet widely implemented in Thailand. This represents a critical gap in regional knowledge. The present study addresses these deficiencies by integrating four distinct efficacy indices—the ACI, POAA, RLS, and ROP—and complementing them with histopathological analysis. By evaluating *E. acervulina*, *E. maxima*, and *E. tenella* isolates from commercial broiler farms, this research aims to determine the current sensitivity of field strains to commonly used anticoccidials. These findings provide essential data to refine shuttle program strategies and strengthen coccidiosis control measures in the Thai poultry sector.

## 2. Materials and Methods

### 2.1. Ethics Statements

All experimental procedures were conducted in accordance with the ARRIVE guidelines, and the current study protocol was approved by the Institutional Animal Care and Use Committee of Rajamanggala University of Technology Tawan‐Ok (Approved number: RMUTTO‐ACUC‐2‐2023‐012). For euthanasia, intravenous sodium pentobarbital was administered to the chicks at a dose of 30 mg/kg body weight to alleviate suffering, subsequently followed by manual cervical dislocation. This procedure was executed by individuals possessing verified technical proficiency, in accordance with the methodology outlined by Zander [15].

### 2.2. Sample Collection

The *Eimeria* spp. isolates used in this study were derived from pooled fecal samples collected from multiple commercial broiler farms across eastern Thailand in December 2022. This region was targeted because it has the highest broiler production density in the country, making it a critical indicator of national coccidiosis trends. Samples were collected aseptically by gathering 60 g of fresh feces per house using a semirandomized approach to ensure representative sampling and reduce bias. Five samples were placed into sterile plastic bags with about 300 g of feces and thoroughly mixed. They were then transported to the alternative to antibiotics (ATA) Research Unit at Rajamangala University of Technology, Tawan‐Ok. The collected fecal material was transferred into 250 mL containers containing a 2.5% potassium dichromate solution. To facilitate oocyst sporulation and ensure viability, the solution was aerated and maintained at 37°C for 48 h. Following sporulation, the samples underwent a rigorous identification process. Species identification was initially conducted based on morphological characteristics, including oocyst shape and size, as outlined by Long and Reid [16]. To ensure diagnostic precision, these morphological findings were further validated using a molecular approach. Genomic DNA was extracted from the purified oocysts, and species confirmation was performed via a multiplex PCR assay. This assay utilized seven species‐specific primer pairs targeting the internal transcribed spacer 1 (ITS1) region of *Eimeria* spp., following the protocol described by Haug et al. [17].

### 2.3. Purification and Inoculum Doses for Experimental Infection Assessment

For purification, approximately 100 oocysts of each *Eimeria* species were collected using the single oocyst separation technique (Method 1) as previously described [18]. To evaluate the virulence and dosage of *Eimeria* before AST, a total of forty‐five coccidia‐free 1‐day‐old broiler chickens were raised until they reached 14 days of age. The chickens, which had similar weights, were randomly assigned to 9 groups of 5 each. Groups 1, 2, 3, and 4 received oral inoculations of diluted *E. acervulina*, whereas Groups 5, 6, 7, and 8 received oral inoculations of diluted *E. maxima*. The specific *Eimeria* species present in each of the four inocula are detailed in Table 1, and a blank control group was established for comparison. At 7 days post‐inoculation (DPI), the birds were weighed individually and then euthanized by cervical dislocation for necropsy and lesion scoring. The inoculation doses were calibrated to achieve a coccidial lesion score of 2–3 while ensuring no mortality and a 15%–25% reduction in weight gain in infected, unmedicated birds [19]. For *E. tenella*, we adjust the inoculum doses based on our previous study [20].

**TABLE 1:** Infective dose inoculated in each group.

Treatment	Inoculated infective dose
1	*E. acervulina*: 31,250 oocysts/bird
2	*E. acervulina*: 62,500 oocysts/bird
3	*E. acervulina*: 125,000 oocysts/bird
4	*E. acervulina*: 250,000 oocysts/bird
5	*E. maxima*: 5000 oocysts/bird
6	*E. maxima*: 10,000 oocysts/bird
7	*E. maxima*: 20,000 oocysts/bird
8	*E. maxima*: 40,000 oocysts/bird
9	Non‐infected

### 2.4. Anticoccidial Drug Sensitivity Test

A total of 112 one‐day‐old male Ross 308 broiler chickens were randomly allocated into four groups, each with equal average weights. Each group comprised four replicates of seven birds. The sample size was determined prior to the experiment using G∗Power 3.1.9.7 software. Based on a power analysis, a total sample size of 112 birds was identified as the minimum required to achieve 80% power (1‐β) at an alpha level of 0.05, ensuring sufficient sensitivity to detect significant differences in performance and lesion scores between groups. The dietary composition was tailored to support the birds’ growth stage, as outlined in Supporting Table 1, and complied with the NRC requirements [21]. The birds had free access to filtered water and feed. The feed ingredients were blended with commercial premixes containing anticoccidial agents to formulate experimental rations at the required drug concentrations. These anticoccidials were incorporated into the starter, grower, and finisher diets at the specified levels throughout the study. Any uneaten feed was weighed and disposed of weekly. The chickens were housed in heat‐treated battery pens with wire flooring, occupying a total surface area of 2 m^2^ within a coccidia‐free environment. The temperature in the pens was maintained between 29°C and 32°C using electric heating provided by 100W bulbs. Additionally, red light was continuously supplied for 24 h throughout the experimental period. The organization of the groups was as follows: (1) The uninfected‐untreated control (UUC, T1) group, which received the basal diet without *Eimeria* inoculation; (2) The infected‐untreated control (IUC, T2) group, which received the basal diet and was inoculated with *Eimeria*; (3) The T3 group, which was supplemented with 40 ppm nicarbazin combined with 40 ppm narasin per ton of starter feed and 70 ppm salinomycin in the grower feed; and (4) The T4 group, which was supplemented with 40 ppm nicarbazin combined with 40 ppm narasin per ton of starter feed and five ppm maduramicin in the grower feed, as indicated in Table 2. All groups were provided an unmedicated basal diet for the finisher feed until the experiment’s conclusion. At 21 days, all chickens were weighed to record their initial body weight and leg‐tagged for identification. Subsequently, the chickens were orally inoculated with a suspension containing 2 × 10^5^ sporulated oocysts of *E. acervulina*, 1 × 10^5^ oocysts of *E. maxima*, and 1.5 × 10^4^ oocysts of *E. tenella*, administered in a 1 mL volume directly into the crop. Meanwhile, the unmedicated control groups remained uninfected and were given distilled water instead. The birds were monitored daily for mortality, and any that died were collected for necropsy and weight measurement. Fecal samples were collected from all chickens 6 DPI, and the average number of OPG of feces was calculated for each replicate. The samples were diluted in saturated NaCl, and oocysts were counted microscopically using a McMaster counting chamber [13]. At seven days post‐infection, four chickens from each replicate (16 chickens per group) were weighed and euthanized by cervical dislocation for intestinal sampling. Immediately after euthanasia, the intestinal lesion scores were evaluated in a blinded manner by assessors using previously established scoring techniques [19]. For all necropsied intestinal samples, 3‐cm sections were collected from the duodenum, jejunum–ileum, and cecum’s central regions. These samples were rinsed with phosphate‐buffered saline and then fixed in 10% formalin. The fixed tissues were subsequently embedded in paraffin and sectioned at 4 μm for staining with hematoxylin and eosin. Images of the prepared slides were captured with a light microscope equipped with a camera. The study focused on visible schizonts or gametocytes, which indicated the invasion of epithelial cells [22]. For quantitative analysis, the prevalence of infection was calculated as the percentage of birds positive for these endogenous stages in each treatment group (*n* = 16). Each intestinal segment (duodenum, jejunum–ileum, and cecum) was evaluated for the presence or absence of parasites. At the end of the experiment, the remaining chickens were weighed to calculate weight gain and then euthanized. Chickens that died during the study were assigned a score of 4.

**TABLE 2:** Experimental design for anticoccidial sensitivity test.

Treatment^1^	Coccidiostat dose (ppm)		*Eimeria* challenge
	Starter (1–10 days)	Grower (11–28 days)	
T1, UUC	No	No	No
T2, IUC	No	No	Yes
T3	NAS + NIC (40 + 40)	SAL (70)	Yes
T4	NAS + NIC (40 + 40)	MAD (5)	Yes

*Note:* NAS = narasin; NIC = nicarbazin; SAL = salinomycin; MAD = maduramicin.

Abbreviations: IUC, infected‐untreated control; ppm, part per million; UUC, uninfected‐untreated control.

^1^Experimental group of 4 replicates with 7 individuals each.

### 2.5. Evaluation of Drug Resistance

The drug resistance of *Eimeria* spp. was evaluated using a combination of four indices: the POAA, ROP, the ACI, and RLS. The POAA was calculated using weight and mortality data, following the formula: POAA = (GSR in the medicated group − GSR in the infected‐unmedicated group)/(GSR in the uninfected‐unmedicated group − GSR in the infected‐unmedicated group) × 100%. Here, GSR (growth and survival ratio) is defined as the final body weight divided by the initial body weight. A POAA greater than 50% indicated sensitivity, while a POAA of 50% or less was classified as resistance [8]. The ROP value was calculated as previously outlined [10] using the formula: ROP = (oocyst output of the treated and infected group ÷ oocyst output of the positive control group) × 100. An ROP value of less than 15% indicated sensitivity, while a 15% or greater value indicated resistance. The RLS was calculated using the following formula: RLS = (average lesion score in the infected‐unmedicated group − average lesion score in the medicated group)/average lesion score in the infected‐unmedicated group × 100%. An RLS of 50% or greater indicated sensitivity, while an RLS of less than 50% was classified as resistance [23]. The ACI was calculated using the formula: ACI = (rate of relative body weight gain + survival rate) − (lesion score + oocyst value). The oocyst value was determined: oocyst value = (number of fecal oocysts in the treated‐infected group)/(number of fecal oocysts in the infected untreated control group) × 100%. An ACI value of 160 or greater indicated sensitivity, while an ACI value of less than 160 represented resistance [24]. To provide a robust and integrated evaluation of drug efficacy, a cumulative point‐based classification system was employed, as described by Flores et al. [25]. This multidimensional approach was specifically selected to mitigate the risk of misinterpretation arising from discordant results among individual indices, in which an isolate might show parasitological sensitivity (e.g., low oocyst production) but clinical resistance (e.g., persistent intestinal lesions). Each of the four indices (ACI, POAA, RLS, and ROP) was evaluated independently against its respective resistance threshold. The overall resistance status of each *Eimeria* strain was then determined by the number of indices indicating resistance: a strain was classified as “severely drug‐resistant” if three or four indices met the resistance criteria, “moderately resistant” if two indices indicated resistance, and “slightly drug‐resistant” if only one index showed resistance. The absence of resistance across all four indices signified that the strain remained “sensitive” to the treatment. This integrated scoring system ensures a balanced assessment by incorporating both performance‐based and parasitological parameters into the final classification.

### 2.6. Statistical Analysis

Statistical analyses were conducted using GraphPad Prism 8. BWG, lesion scores, and oocyst production were assessed using one‐way ANOVA followed by Dunnett’s multiple comparison tests. Additionally, the frequency of parasite detection in histopathological samples was compared between groups using Fisher’s exact test. Results are presented as means ± standard error of the mean. A *p* value of less than 0.05 was considered statistically significant. It is acknowledged that, although intestinal lesion scores are inherently ordinal, they were analyzed as continuous variables, following long‐standing conventions in coccidiosis research [19], to enable standardized comparisons across treatment groups.

## 3. Results

### 3.1. Dose Titration Trial

Four doses of each isolate were used to assess pathogenicity. Among the two *Eimeria* species, no mortality was observed in the groups infected with either *E. acervulina* or *E. maxima*. The average lesion scores in chickens infected with *E. acervulina* showed a successive decline, alongside a corresponding increase in average weight gain (g). However, the highest dose of *E. maxima* did not result in a lesion score of 2. In summary, the inoculation doses for *E. acervulina* and *E. maxima* used in this study were 200,000 and 100,000 oocysts per bird, respectively.

### 3.2. Drug Sensitivity

#### 3.2.1. Growth Performance Results

No coccidiosis‐related mortality was observed during this trial. The T1 group exhibited the highest weight gain, while the weight gain of the T2 group at 28 and 35 days of age was significantly lower (*p* < 0.05) when compared to the T1 group (Table 3). Infected birds treated with salinomycin and maduramicin demonstrated significantly greater weight gain than the T2 birds, with no significant difference in weight gain compared to the T1 birds on Days 21–28 and 21–35. Additionally, there were no notable differences between the salinomycin and maduramicin treatment groups. Following this, all POAA values were calculated according to the GSR protocol. POAA values below 50% indicated partial resistance to weight‐gain recovery for both coccidiostats tested; notably, resistance to maduramicin was numerically higher on this metric.

**TABLE 3:** Body weight gains and percent of optimum anticoccidial activity (POAA, %).

Groups	Weight gain^1^ (g) 21–28 days	GSR	POAA (%)	Weight gain^1^ (g) 21–35 days
T1	593.39 ± 13.34^a^	1.64	—	1155.32 ± 31.73^a^
T2	399.28 ± 12.22^b^	1.41	—	616.46 ± 32.34^b^
T3	497.00 ± 12.26^cd^	1.50	38.66	905.08 ± 31.11^cd^
T4	464.42 ± 8.65^d^	1.44	13.33	900.84 ± 23.05^d^

*Note:* POAA > 50% indicates sensitivity; POAA ≤ 50% indicates resistance.

Abbreviation: GSR, growth survival ratio.

^1^The values are represented as the mean ± SEM (*n* = 28 per group).

^a,b,c,d^Superscripts within a column indicate significant differences (*p* < 0.05).

### 3.3. Lesion Scores

Intestinal macroscopic lesions were completely absent in the T1 group of birds, while they were present in the T2 group, with substantially higher lesion scores observed in the latter. Overall, birds treated with anticoccidial drugs exhibited significantly lower intestinal lesion scores (*p* < 0.05) compared to those in the T2 group, except in the cecal region of the T3 group (Table 4). The ordinal score trends for birds supplemented with salinomycin indicated that this drug was effective in preventing lesions caused by *E. acervulina* and *E. maxima* but was ineffective against *E. tenella* infection. In contrast, the ordinal score trends for birds treated with maduramicin suggested that the drug successfully suppressed lesions from all three *Eimeria* species. Furthermore, maduramicin was effective in reducing lesions associated with *E. maxima*, as evidenced by a RLS. Between the two drugs, both anticoccidial agents significantly reduced lesion occurrence in the mid‐intestinal regions; however, their efficacy was not statistically significant in the upper intestinal and cecal regions. While mean lesion scores were analyzed as continuous data, consistent with industry convention, the results demonstrated that maduramicin provided broader clinical protection across intestinal segments than salinomycin.

**TABLE 4:** Gross lesion score (LS) in the duodenum, mid‐intestine, and ceca, reduction of lesion scores (RLS, %), and total mean lesion score (TMLS) at 7 dpi.

Groups	Duodenum		Mid‐intestine		Ceca		TMLS
	LS^1^	RLS (%)	LS^1^	RLS (%)	LS^1^	RLS (%)	
T1	0^a^	—	0^a^	—	0^a^	0	0
T2	2.66 ± 0.12^b^	—	2.40 ± 0.13^b^	—	2.40 ± 0.23^b^	—	7.46
T3	1.66 ± 0.15^c^	37.50	1.53 ± 0.13^c^	36.11	1.73 ± 0.22^bc^	27.77	4.93
T4	1.53 ± 0.13^c^	42.50	1.00 ± 0.13^d^	58.33	1.60 ± 0.18^c^	33.33	4.13

*Note:* RLS interpretation: sensitive ≥ 50%; resistant < 50%.

^1^The values are represented as the mean ± SEM (*n* = 16 per group).

^a,b,c,d^Superscripts within a column indicate significant differences (*p* < 0.05).

### 3.4. Fecal Oocyst Shedding

No oocysts were detected in the T1 groups. Among the medicated cohorts, the group treated with salinomycin exhibited a higher fecal oocyst count than the maduramicin group (Table 5). Overall, fecal oocyst counts showed a significant decrease (*p* < 0.05) in both the salinomycin and maduramicin groups when compared to the T2 group. Our field isolates demonstrated relative sensitivity to the drugs salinomycin and maduramicin, as indicated by the ROP values.

**TABLE 5:** The average oocyst production and relative oocyst production (%ROP) results.

Groups	OPG^1^ × 10^3^	ROP (%)
T1	0^a^	—
T2	44.74 × 5.52^b^	—
T3	4.37 × 0.52^c^	9.77
T4	3.89 × 0.52^c^	8.17

*Note:* OPG: Oocysts per gram of feces; ROP less than 15% indicates sensitivity; 15% or more indicates resistance.

^1^The values are represented as the mean ± SEM (*n* = 28 samples per group).

^a,b,c^Superscripts within a column indicate significant differences (*p* < 0.05).

### 3.5. The ACI and Overall Judgment of Drug Resistance

The assessment of drug sensitivity revealed discordant results across individual indices: while ACI values (> 160) suggested effectiveness in supporting overall productivity, POAA values (< 50%) indicated resistance to growth performance (Table 6). This integrated analysis led to the final classification of the field isolates as “moderately resistant” to both anticoccidial agents, accurately reflecting the partial loss of efficacy observed across different parameters.

**TABLE 6:** Results for the anticoccidial index (ACI).

Groups	Survival rate (%)	Body weight gain rate (%)	Lesion scores^1^	Oocyst value	ACI
T1	100	100	0	0	200
T2	100	67.26	7.46	100	59.80
T3	100	83.75	4.93	9.77	169.05
T4	100	78.26	4.13	8.17	165.96

^1^Total mean lesion score (TMLS): an ACI ≥ 160 indicates sensitivity; less than 160 indicates resistance.

### 3.6. Histopathological Analysis

**FIGURE 1:**
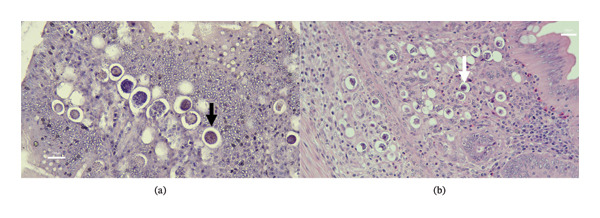
Histopathological characterization of *Eimeria* spp. in the lamina propria. Jejunum (a); cecum (b); macrogametocytes (black arrows); oocyst (white arrow).

No *Eimeria* schizonts or gametocytes were detected in the T1 groups (0/16). In contrast, the T2 group presented evidence of *E. acervulina*, *E. maxima*, and *E. tenella*, with organisms identified in all segments (14/16) (Figure 1). Quantitative analysis of infection prevalence revealed that the salinomycin group had a higher frequency of detectable parasite stages (13/16, 81.3%) than the maduramicin group (10/16, 62.5%). However, Fisher’s exact test indicated that these differences in prevalence among the infected groups were not statistically significant (*p* > 0.05), suggesting that although both drugs reduced lesion severity, neither fully eliminated epithelial invasion.

## 4. Discussion

In this study, the AST was designed to simulate the common shuttle programs used in Thai poultry production, which typically transition from synthetic compounds to ionophores to mitigate the risk of resistance [26]. Our findings demonstrate that while salinomycin and maduramicin—both polyether ionophores—disrupt the parasite’s ion gradients [27], they provided only partial protection against the current field isolates. To the best of our knowledge, this study provides the first AST‐based evidence of moderate resistance in *Eimeria* field isolates derived from the Thai shuttle program. While previous reports have documented general resistance in the region, our findings uniquely demonstrate how these isolates maintain clinical survival (high ACI) while exhibiting significant growth depression (low POAA) under simulated field conditions. The shuttle program design in this study—utilizing medicated starter and grower diets followed by a nonmedicated finisher phase—directly mirrors the standard commercial practices in Thailand. This “drug‐free” finisher phase is strictly implemented across the Thai poultry industry to ensure compliance with drug withdrawal period requirements before slaughter. It is important to emphasize that nearly all commercial broiler operations in Thailand use shuttle programs exclusively as the primary strategy for controlling coccidiosis. Consequently, evaluating efficacy within this specific framework is more regionally relevant than comparing it with nonshuttle or vaccine‐only systems, which are not currently the industry standard. Furthermore, while this study focused exclusively on a shuttle framework, the experimental design was intended to evaluate the efficacy of rotating different ionophores within the grower phase. This approach aligns with recent global field research, in which the focus has shifted toward optimizing the rotation of specific combinations of chemicals and ionophores. For instance, Dehaeck et al. [28] reported that implementing a “chemical break” within a long‐term shuttle program significantly improved the feed conversion rate (FCR) from 1.73 to 1.66 (*p* < 0.001). By focusing on internal comparisons of different grower‐phase medications, this study provides actionable data to refine the shuttle protocols that dominate Thai poultry production. As evidenced by recent European data, such optimizations are the primary drivers of both economic profitability and sustainability in modern, high‐density broiler systems. While several authors raised concerns about resistance in the years following its introduction, comprehensive research on this issue remains limited. This study used AST simulation to model practical applications and demonstrated that anticoccidials are commonly used in shuttle programs in Thailand. Birds administered salinomycin or maduramicin exhibited significantly greater weight gain than the T2 group; however, their weight gain remained lower than that of the T1 group, suggesting that these treatments were only partially effective. The mode of action of salinomycin involves the destruction of sporozoites and the inhibition or significant delay of schizont maturation, which occurs 30–72 h after ingestion of oocysts [29]. Maduramicin facilitates the transport of cations across cell membranes, thereby influencing various processes that rely on ion movement. Ultimately, the accumulation of sodium ions within the parasite induces osmotic water influx, leading to parasite swelling and eventual rupture [27]. As evidenced by previous studies, the enhanced efficacy of maduramicin against ionophore‐tolerant *Eimeria* is associated with reduced lesions and mortality, as well as improved broiler performance [30]. According to the POAA index, the field isolates were resistant to salinomycin and maduramicin. These findings align with brief reports documenting the resistance of certain coccidial isolates to these drugs [31, 32]. Consequently, they suggest that coccidiosis can still diminish production efficiency in infected individuals despite prophylactic anticoccidial drug administration in birds [8]. This underscores the economic significance of chicken coccidiosis, which can be attributed to two primary factors. First, the diminishing efficacy of commonly used ionophores, such as salinomycin and maduramicin, is primarily due to the emergence of acquired drug resistance in coccidial isolates. This process arises from genetic mutations and intense selective pressure—factors exacerbated by long‐term suboptimal dosing and the frequent use of similar ionophore classes in shuttle or rotation programs across Thailand [33]. As these field isolates have likely been exposed to multiple ionophores over successive generations, the survival and proliferation of tolerant subpopulations have culminated in the “moderate resistance” status observed in this study. Such findings highlight an urgent need for diversifying control strategies, such as integrating synthetic chemicals or vaccination, to break the cycle of ionophore cross‐resistance [34]. Second, beyond acquired resistance, inherent limitations in drug efficacy across intestinal segments further affect overall control. Our results indicate that salinomycin did not significantly reduce lesions in the cecum (*E. tenella*) compared to the nonmedicated treatment. This reflects the well‐documented inability of certain ionophores to completely prevent severe infections in specific regions, particularly the ceca, as evidenced by decreased sensitivity in lesion scores [35]. Unlike acquired resistance, this failure may also stem from suboptimal drug distribution or improper management practices that prevent the medication from reaching therapeutic levels in the distal gut. Such incomplete parasite eradication allows subclinical infections to persist, continuing to negatively affect growth and productivity despite prophylaxis. Although lesion scores are inherently ordinal, their statistical analysis in this study followed established industry conventions to ensure results remain comparable with such foundational research. Furthermore, salinomycin failed to decrease lesions in any region, as determined by the RLS. In contrast, maduramicin was able to reduce lesions in the mid‐intestine (*E. maxima*). This finding is consistent with previous studies [36] that report varying degrees of resistance to anticoccidial drugs, attributable to the diverse strategies employed in managing coccidiosis and the availability of different drugs. The findings on oocyst production revealed a significant anticoccidial effect of salinomycin and maduramicin compared with the nonmedicated group. Our results are consistent with those of a similar study conducted in Pakistan [31]. A comprehensive evaluation of the drug sensitivity of our field isolates, as assessed by the ACI, indicated sensitivity to salinomycin and maduramicin. These findings stand in stark contrast to a related study conducted in Iran, which reported significant resistance to these drugs [8]. The variations observed in reported drug sensitivity results for poultry *Eimeria* may be attributed to differences among isolates in their responses to the same or similar anticoccidial drugs. The salinomycin treatment still showed a high prevalence of *Eimeria* in 13 of 16 cases, slightly lower than in the untreated control, but indicating that *Eimeria* species remain prevalent despite treatment. This suggests possible resistance of the salinomycin‐treated strain to the challenge strain under the conditions tested. The higher prevalence in the salinomycin‐treated group than in the maduramicin group suggests that the latter may be more effective. These results are consistent with previous reports concerning the sensitivity of field isolates of *Eimeria* species, particularly *E. acervulina* [37]. However, they contrast with findings from other studies involving mixed *Eimeria* species infections [38], which demonstrated that the sensitivity levels of *Eimeria* spp. are strongly dependent on the specific strains or species present in field populations. The observed resistance to both salinomycin and maduramicin in our field isolates suggests cross‐resistance within the ionophore class. Although these drugs have different cation affinities (monovalent for salinomycin vs. monovalent/divalent for maduramicin), their shared mechanism of disrupting ion transport across parasite membranes often leads to overlapping resistance profiles after prolonged field exposure [34]. The partial efficacy observed—characterized by maintained clinical survival (high ACI) but reduced growth performance (low POAA)—further indicates tolerance to ionophores. This phenomenon likely stems from the parasites’ ability to compensate for ion imbalances, possibly through enhanced sodium–potassium pump activity or changes in membrane lipid composition, allowing them to survive and replicate despite the presence of the drug [39]. The moderate resistance observed in this study underscores the need to transition from sole reliance on anticoccidial drugs to integrated control strategies. Our findings suggest that traditional ionophore programs are becoming less effective, highlighting the potential for Bio‐shuttle programs as a sustainable alternative. As demonstrated by N’Guetta et al. [40], a “3‐way strategy”—combining immune modulators (such as beta‐glucans), probiotics for microbiome management, and botanical compounds—can effectively complement or even replace conventional anticoccidials. In a bio‐shuttle context, the use of live vaccines enables controlled oocyst circulation, which stimulates the bird’s natural immunity, while supplementary strategies address secondary challenges such as *Clostridium perfringens*. Integrating such approaches would reduce ionophore selection pressure, potentially restoring the efficacy of these drugs for future use while maintaining broiler performance and intestinal health. This integrated shift is crucial for mitigating the cross‐resistance cycle identified in our field isolates. The observed differences between treatments underscore the necessity for continuous monitoring and the strategic rotation of anticoccidial agents. However, it must be acknowledged that this study possesses certain limitations. Anticoccidial resistance is highly farm‐specific and is profoundly influenced by localized factors, including specific drug histories, biosecurity protocols, litter management practices, and stocking densities. Consequently, while our findings from eastern Thailand provide a critical snapshot of resistance within high‐density production systems, they should be interpreted as an indicative case study rather than a universal trend across all Thai poultry operations. Future research should encompass a broader geographical range and longitudinal data to account for these farm‐level variables. Furthermore, the development of alternative strategies, such as integrating natural agents like organic acids or essential oils, remains essential for mitigating selection pressure and improving the effectiveness of long‐term control amid evolving resistance.

## 5. Conclusion

By employing four comprehensive efficacy indices, this study provides the first AST‐based evidence of moderate resistance in *Eimeria* field isolates derived from the Thai shuttle program. While these isolates maintain clinical survival (high ACI), the significant growth depression (low POAA) identifies a critical loss in production efficiency. To address this, routine AST monitoring and the integration of rotation or vaccination strategies are urgently needed in Thai broiler farms. Specifically, the implementation of bio‐shuttle programs—combining live vaccines with targeted anticoccidial rotation—is recommended to mitigate selection pressure and displace resistant field strains. These actionable steps are essential to ensure long‐term productivity and sustainable coccidiosis management amid evolving drug resistance.

## Funding

This research was supported by the Office of the Permanent Secretary, Ministry of Higher Education, Science, Research and Innovation (OPS MHESI), Thailand Science Research and Innovation (TSRI) (Grant No. RGNS 65‐091).

## Conflicts of Interest

The authors declare no conflicts of interest.

## Supporting Information

Additional supporting information can be found online in the Supporting Information section.

## Supporting information


**Supporting Information** This section presents the ingredients and nutrient profiles of the basal diets formulated for different experimental phases. The diets include proportions of corn, soybean meal, rice solvent bran, palm oil, minerals, amino acids, and premixes, with detailed ingredient percentages provided for the starter (0–10 days), grower (11–28 days), and finisher (29–35 days) periods. The nutrient contents—such as metabolizable energy, crude protein, fat, fiber, minerals, and vitamins (A, D3, E, K3, B‐complex, biotin)—and trace minerals are also detailed, reflecting formulation adjustments across the growth stages.

## Data Availability

The datasets used and/or analyzed during the current study are available from the corresponding author upon reasonable request.
