# Supplementary material for: Anticoccidial Resistance in Eimeria spp. From Thai Broiler Farms Using Shuttle Programs
Source: Vet Med Int. 2026 May 22;2026:8243240. doi: 10.1155/vmi/8243240 (PMC13197649; doi:10.1155/vmi/8243240)
Supplement: Supplementary file 1 — Supporting Information This section presents the ingredients and nutrient profiles of the basal diets formulated for different experimental phases. The diets include proportions of corn, soybean meal, rice solvent bran, palm oil, minerals, amino acids, and premixes, with detailed ingredient percentages provided for the starter (0–10 days), grower (11–28 days), and finisher (29–35 days) periods. The nutrient contents—such as metabolizable energy, crude protein, fat, fiber, minerals, and vitamins (A, D3, E, K3, B‐complex, biotin)—and trace minerals are also detailed, reflecting formulation adjustments across the growth stages. [file VMI-2026-8243240-s001.docx]

Supplementary Table 1 Ingredients and nutrient contents of basal diets in different experimental periods.

| Ingredients, % | Starter (d 0-10) | Grower (d 11-28) | Finisher (d 29-35) |
| --- | --- | --- | --- |
| Corn | 52.76 | 58.17 | 62.54 |
| Soybean meal, 48% CP | 34.93 | 31.55 | 26.11 |
| Rice solvent bran | 5.92 | 4.86 | 6.32 |
| Palm oil | 2.50 | 2.50 | 2.50 |
| Monodicalciumphosphate21 | 1.11 | 0.67 | 0.33 |
| Calcium carbonate | 1.18 | 0.86 | 0.83 |
| L-Lysine | 0.28 | 0.21 | 0.25 |
| DL-Methionine | 0.38 | 0.33 | 0.31 |
| L-Threonine | 0.11 | 0.06 | 0.06 |
| L-Valine | 0.07 | 0.04 | 0.01 |
| Salt | 0.30 | 0.30 | 0.30 |
| Phytase+NSP enz 100g/t (-70 kcal/kg) | 0.01 | 0.01 | 0.01 |
| Choline Chloride 60% | 0.28 | 0.27 | 0.25 |
| Premix^1^ | 0.18 | 0.18 | 0.18 |
| Total | 100.00 | 100.00 | 100.00 |
| Nutrient contents |  |  |  |
| Metabolizable energy, kcal/kg | 2,975.00 | 3,050.00 | 3,100.00 |
| Crude Protein, % | 23.00 | 21.50 | 19.50 |
| Fat, % | 4.76 | 4.91 | 5.28 |
| Crude Fiber, % | 4.12 | 3.93 | 3.35 |
| Calcium, % | 0.95 | 0.75 | 0.65 |
| Total Phosphorus, % | 0.88 | 0.76 | 0.65 |
| Available phosphorus, % | 0.50 | 0.42 | 0.36 |
| Salt, % | 0.34 | 0.33 | 0.34 |
| Choline, mg/kg. | 1,700.00 | 1,600.00 | 1,500.00 |
| Digestible Lysine, % | 1.32 | 1.18 | 1.08 |
| Digestible Methionine, % | 0.68 | 0.62 | 0.59 |
| Digestible Methionine + Cystine, % | 1.00 | 0.92 | 0.86 |
| Digestible Threonine, % | 0.88 | 0.79 | 0.72 |
| Digestible Tryptophan, % | 0.26 | 0.24 | 0.21 |
| Digestible Valine, % | 1.00 | 0.91 | 0.84 |

^1^Provides each kg of diet: Vit. A: 12000 IU, Vit. D3: 5000 IU, Vit. E: 65.0 mg, Vit. K3: 3.0 mg, Vit. B1: 3.0 mg, Vit. B2: 6.5 mg, Vit. B6: 3.2 mg, Vit. B12: 0.02 mg, Nicotinic acid: 16.0 mg, Folic acid: 1.8 mg, Biotin: 180.0 mg, D-calcium: 16 mg, Copper: 80.0 mg, Iodine: 5.0 mg, Selenium: 100.0 mg, Iron:40.0 mg, Manganese: 80.0 mg, Zinc: 60.0 mg, Cobalt: 100.0 mg.
